# Characteristics of glucose-6-phosphate dehydrogenase mutations in newborns with deficiency from 2021 to 2022 in the Heze area of China

**DOI:** 10.3389/fimmu.2025.1472474

**Published:** 2025-04-17

**Authors:** Xin Zhang, Yanan Duan, Xiao Zhang, Miaomiao Li, Ling Li, Renwei Zhang, Shiguo Liu

**Affiliations:** ^1^ Department of Medical Genetics, Affiliated Hospital of Qingdao University, Qingdao, China; ^2^ Prenatal Diagnosis Center of Qingdao University Affiliated Hospital, Qingdao, China; ^3^ Department of Biochemical Laboratory, Heze Medical College, Heze, China; ^4^ Department of Obstetrics and Gynecology, The Affiliated Hospital of Qingdao University, Qingdao, China; ^5^ Laboratory Department, Heze Municipal Hospital, Heze, China

**Keywords:** G6PD, pathogenic variant, glucose-6-phosphate dehydrogenase deficiency, Heze area, new mutation site

## Abstract

**Introduction:**

Glucose-6-phosphate dehydrogenase (G6PD) deficiency has a distinct regional and ethnic heterogeneity in distribution, and information on the molecular characteristics of G6PD deficiencies in the Heze area, Shandong Province, China, is limited. We aimed to explore the incidence and genetic mutations characteristic of G6PD enzyme deficiencies in newborns in the Heze area to investigate the pathogenicity of new G6PD mutations.

**Methods:**

We measured G6PD activity in 114,285 neonates born in the Heze area and identified 80 patients with G6PD deficiencies. The genetic mutations in G6PD in these patients were analyzed using Sanger sequencing. Functional studies were conducted by constructing eukaryotic expression vectors, transfecting them into HEK-293T and HELA cells, and measuring the mRNA and protein levels and G6PD enzymatic activity.

**Results:**

The incidence of G6PD deficiency in the study population was 0.07% (80/114,285). We identified 17 mutation types with a 100% G6PD mutation detection rate, with four of them being significant: c.479G>A, c.404A>T, and c.486-7C>G being globally novel mutations, while c.682G>A has never been reported in China before. Functional studies revealed that the heterozygous missense mutations c.479G>A/p.S160N and c.404A>T/p.N135I increased mRNA levels, decreased protein expression, and reduced G6PD activity.

**Discussion:**

The incidence of neonatal G6PD deficiency in the Heze area is low, and the most commonly mutated loci were c.1388G>A, c.487G>A, and c.1376G>T. Among these mutations, c.479G>A/p.S160N, and c.404A>T/p.N135I are potentially pathogenic. These mutations may cause G6PD deficiency via different mechanisms, thereby requiring further experimental investigation.

## 1 Introduction

Deficiency of glucose-6-phosphate dehydrogenase (G6PD) is a common inherited red blood cell enzyme deficiency caused by mutations in G6PD, which has an X-linked incompletely dominant inheritance and can cause hemolytic anemia (1). Approximately 500 million individuals globally are impacted by G6PD deficiency, showing a total prevalence rate of approximately 5% (2). While most G6PD deficient patients have no symptoms, untreated hemolytic anemia can be fatal. An average of 4,100 deaths attributable to G6PD deficiency occurred each year from 1990 to 2013 (3).

G6PD is located on chromosome Xq28 and comprises 13 exons (including an initial non-coding exon) and 12 introns, encoding a protein of 515 amino acids (4). G6PD is a regulator of glucose-6-phosphate (G6P) hydrolysis to 6-phosphogluconate (6-PG) and thus controls glycolysis and produces nicotinamide adenine dinucleotide phosphate (NADPH) through the route of pentose phosphate; this is the primary source of NADPH in blood cells. Thus, G6PD mutations can compromise glycolysis, the pentose phosphate pathway, and other metabolic cascades, leading to less energy production, less NADPH generation, more oxidative stress, erythrocyte hemolysis, and various pathologies, including hemolytic anemia and neonatal jaundice.

Mutations in the G6PD gene induce diverse enzyme activity levels. The World Health Organization (WHO) has overhauled the G6PD variant classification, scrapping the old arbitrary-threshold-based system. The novel scheme classifies variants into four classes (A, B, C, U) according to clinical features and median enzyme activity in hemizygous males or homozygous females (5). Class A, with median G6PD activity <20%, is associated with chronic non-spherocytic hemolytic anemia; variants having <20% activity and only acute hemolytic anemia triggered by oxidant drugs, fava beans, or infections are grouped into Class B. Class B (median G6PD activity <45%) encompasses most common polymorphic variants and entails risks of severe neonatal jaundice and acute hemolytic anemia from such stimuli, incorporating former Class II and III. Class C (median G6PD activity >60%) generally does not trigger hemolysis. Class U serves as a provisional classification for variants lacking adequate clinical data.

Notably, approximately 220 defective alleles of G6PD have been identified worldwide, and there is distinct regional and ethnic heterogeneity in their distribution (5–7). The population in Heze area, situated in the southwestern region of Shandong Province in China, is influenced by various factors, including marriage, trade, geography, and the environment, and they may harbor unknown genetic mutations causing G6PD deficiency. However, information on the molecular features of G6PD deficits in this region is limited. In this study, we aimed to provide a resource for the screening, diagnosis, and treatment of G6PD deficiency in newborns in the Heze area by exploring its regional incidence and genetic mutation spectrum.

## 2 Materials and methods

### 2.1 Participants

A total of 114,285 infants (61,246 males and 53,039 females) born between January 2021 and June 2022 in Heze area were enrolled in the study. We used the genetic screening processor (GSP) automatic fluorescence immunoassay analyzer to screen for G6PD enzyme activity, and identified 80 infants with G6PD deficiency (75 males and 5 females), who were then included in further genetic examination. Prior to their involvement in the study, all guardians provided written, informed consent. This study was approved by the Medical Ethics Committee of the Affiliated Hospital of Qingdao University (Approval No. QYFYWZLL27063).

### 2.2 Checking for the absence of G6PD

The newborns were breastfed at least six times before blood collection; the blood samples were collected from the newborns’ heels within 72 h to 7 days of birth, and dropped onto filter paper. The blood samples were air-dried, frozen, and sealed at -20°C before being sent to the Heze Maternal and Child Health Hospital Neonatal Disease Screening and Diagnosis Center. G6PD enzyme activity was measured using a GSP Automated Fluorescence Immunoanalyzer (reference range ≥27 U/gHb). For patients whose G6PD activity was <27 U/gHb, the family was notified, and a new blood sample was collected for retesting. Subsequently, patients whose G6PD activity remained <27 U/gHb after three re-examinations 1 week apart were diagnosed with G6PD deficiency.

### 2.3 DNA extraction and mutation detection from dry blood spot specimens

We extracted genomic DNA from dry blood spot samples, following the instructions of the Tiangen Biological DP334 DNA Extraction Kit (Centrifuge Column). Based on the National Center for Biotechnology Information (NCBI) reference NM_001360016.2, nine primers were designed to cover the 13 exons of G6PD using Primer Express 3.0. To boost efficiency and accuracy, we applied a composite design to four primer pairs, covering exons 3–4, 6–7, 9–10, and 11–12. This method guarantees comprehensive amplification, conserves resources, and minimizes experimental time.

### 2.4 Cell culture

HEK-293T and HELA cells were cultivated in Dulbecco’s Modified Eagle Medium, which contained 10% fetal bovine serum (100 U/mL penicillin and 100 μg/mL streptomycin), at 37°C and 5% CO_2_. All experiments were performed under sterile conditions. To ensure this, disposable straws, 15 mL and 50 mL centrifuge tubes, and cryopreservation tubes were UV-irradiated for 30 min before use, and all reagents were sprayed with 75% ethanol before being placed on the sterilized working surface. Pipettes with different capacities and gun heads were autoclaved before use.

### 2.5 Plasmid construction and cell transfection

Total RNA was isolated from the gallbladder tissues and reverse-transcribed to obtain cDNA. After agarose gel electrophoresis, products from the polymerase chain reaction (PCR) were purified, and reverse PCR amplification was performed to obtain a linearized vector. G6PD was inserted into the pEGFP-N1 expression vector through homologous recombination, and the recombinant product was transformed, recovered, and sequenced to confirm the successful construction of the wild-type (WT) G6PD cloning vector. Primers designed with Primer Premier 5.0 were accustomed to induce the G6PD c.479G>A/p.S160N and c.404A>T/p.N135I mutation using a point mutation kit. DNA detection was performed using agarose gel electrophoresis, which showed clear single bands for the 1548 nucleotide amplified product. A DNA sequencing company verified the mutated gene sequence. One day before transfection, cells were seeded in 12- or 24-well plates. Lip3000 transfection was performed after 18–24 h when the cells reached 80% confluence.

### 2.6 Total RNA isolation and fluorescence quantitative PCR analysis

Total RNA was collected from the transfected cells 24 hours after transfection. The RNA concentration was >300 ng/μL, with a purity of 1.9–2.1. Reverse transcription was performed to obtain cDNA; 20 μL of the cDNA product was then added to 80 μL of ultrapure water and diluted five times. The qPCR primer sequences were 5′-GCTGACATCGCAAACAGGT-3′ (upstream) and 5′-GGCATCATCATGTGGCTTGTTGA-3′ (downstream), using glyceraldehyde phosphate dehydrogenase (GAPDH) as the level reference and primer sequences 5′-CATGTTCGTCATGGTGGAA-3′ (upstream) and 5′-GGCATGGACTGTGGTCGAG-3′ (downstream).

The fluorescence qPCR reaction contained 5 μL cDNA, 0.4 μL upstream primer, 0.4 μL downstream primer, 10 μL 2× ChamQ Universal SYBR qPCR Master Mix, and ddH_2_O to achieve a total volume of 20 μL. The PCR protocol was designed to include 30 seconds of pre-denaturation at 95°C, 40 cycles of denaturation at 95°C for 10 seconds, 30 seconds of annealing at 60°C, 15 seconds of extension at 95°C, 60 seconds of final annealing at 60°C, and 15 seconds of final extension at 95°C. Relative quantification was performed using the 2^−ΔΔCt^ method to analyze G6PD mRNA levels in the different transfection samples.

### 2.7 Western blot analysis of G6PD protein levels

When the cells had grown to a density of 80% to 90%, they were removed from the 12-well plate, the antiquated cultural medium was eliminated, and the cells underwent washing twice with pre-cooled 1× phosphate-buffered saline. We then added 3–5 times the cell volume of pre-cooled newly prepared cell lysis buffer (RIPA: PMSF=100:1) for cell precipitation, gently mixing to ensure that the cells adhering to the bottom of the plate were covered, and the mixture was placed on ice for 30 min. After a complete cell lysis, the solution was moved to a 1.5-mL microfuge tube and centrifuged at 12,000 rpm for 20 min at 4°C. We transferred 44 μL of the supernatant to a new 1.5 mL microfuge tube, measured the protein concentration, and stored the samples in a -80°C freezer. The protein concentration was determined in a 96-well enzyme-labeled plate according to the manufacturer’s instructions. The protein sample (40 μL) was included in 10 μL of 5× loading buffer, mixed well, heated at 100°C for 10 min, and allowed to chill on ice. The consequence sample was used directly for electrophoresis or stored at -20°C.

### 2.8 Analysis of the catalytic activity of G6PD

The WST-8 G6PDH Activity Assay Kit was utilized to assess G6PD enzyme activity in various samples. In this assay, G6P is oxidized to 6-PG, reducing NADP^+^ to NADPH, which subsequently reacts with 1-mPMS to produce orange-yellow formazan, detectable at approximately 450 nm. This method specifically quantifies NADPH generated by G6PDH activity, as it relies on the unique catalytic reaction of the enzyme, enhancing specificity. Control wells are incorporated to eliminate interference from endogenous NADPH, ensuring accurate measurement of G6PDH activity based on its enzymatic pathway.

### 2.9 Statistical analysis

Statistical analysis was employed using SPSS version 26.0 (SPSS Inc., Chicago, IL). Intergroup comparisons were performed using the Chi-squared test for count data and t-test for metric data. Statistical significance was established at P<0.05.

## 3 Results

### 3.1 Incidence of G6PD deficiency in newborns in the Heze area

Among the 114,285 newborns included in the study, 80 (75 males [93.75%] and 5 females [6.25%]) were diagnosed with G6PD deficiency, showing a total incidence rate of 0.07% (80/114,285). The neonatal G6PD deficiency incidence rates in various counties and districts in the Heze area are shown in **Table 1**. Based on the WHO classification for G6PG deficiency, 50/80 (62.50%) of the patients were classified as Class B, 19/80 (23.75%) as Class C, and 11/80 (13.75%) as Class A (**Table 2**).

**Table 1:** Screening of neonatal G6PD deficiency in various counties and districts of the Heze area.

Region	Number of confirmed cases	Number of screened patients	Incidence rate (%)
Yuncheng County	17	12,715	0.13
Cao County	16	16,295	0.10
Juancheng County	10	9,882	0.10
Chengwu County	4	5,470	0.07
Dongming County	6	8,743	0.07
Dingtao District	4	5,989	0.07
Mudan District	13	20,190	0.06
Shan County	4	12,998	0.03
Juye Prefecture	3	11,381	0.03
Development Zone	2	5,927	0.03
High tech Zone	1	4,695	0.02
Total	80	114,285	0.07

G6PD, glucose-6-phosphate dehydrogenase.

**Table 2:** WHO classifications of 80 confirmed children with G6PD deficiency in the Heze area.

WHO classification	Number of confirmed cases	Average G6PD enzyme activity (U/gHb)	Proportion (%)
A	11	3.78	13.75
B	50	9.22	62.50
C	19	20.58	23.75
Total	80		100

The WHO activity ranges for G6PD were defined as Class A <5.4 U/gHb, Class B 5.4–16.2 U/gHb, and Class C >16.2 U/gHb.

WHO, World Health Organization; G6PD, glucose-6-phosphate dehydrogenase.

### 3.2 Genetic mutations in neonatal G6PD deficiencies in the Heze area

**Figure 1:**
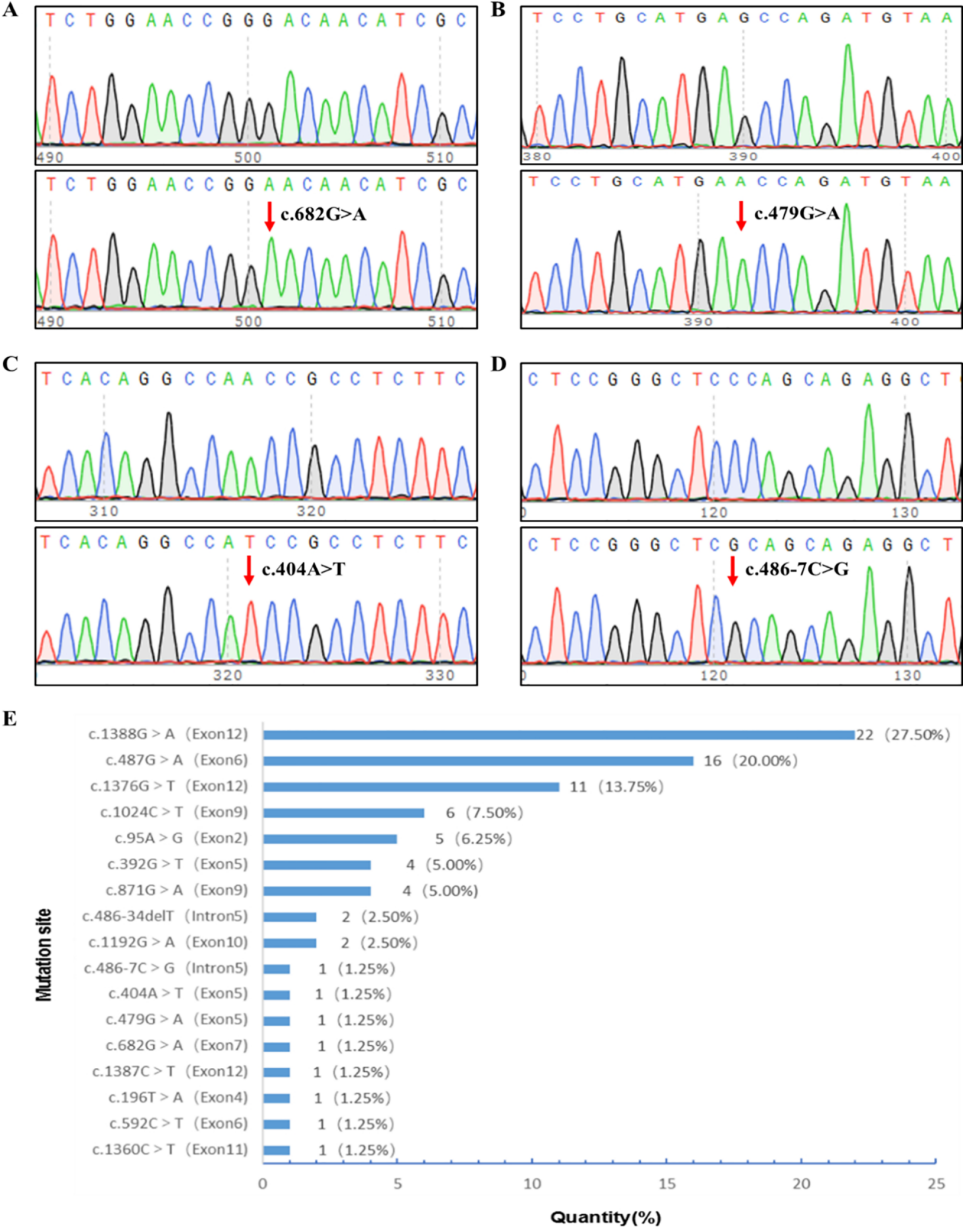
G6PD mutational site sequencing and screening site distribution. **(A)** c.682G>A hemizygotic mutant. **(B)** c.479G>A hemizygotic mutant. **(C)** c.404A>T hemizygotic mutant. **(D)** c.486-7C>G hemizygotic mutant. **(E)** Distribution of screening sites for G6PD mutations in newborns in the Heze area. G6PD, glucose-6-phosphate dehydrogenase.

Upon analyzing all exons of G6PD in the 80 patients with G6PD deficiency, 17 mutation types were detected with a 100% detection rate. Among these, 13 had previously been noted in the Chinese community: c.1388G>A/p.R463H, c.487G>A/p.G163S, c.1376G>T/p.R459L, c.95A>G/p.H32R, c.1024C>T/p.L342F, c.871G>A/p.V291M, c.392G>T/p.G131V, c.1192G>A/p.E398K, c.486-34delT, c.1360C>T/p.R454C, c.592C>T/p.R198C, c.196T>A/p.F66I, and c.1387C>T/p.R463C. Upon searching the MasterMind, NCBI ClinVar, and the National Genome Database of China, we identified four novel mutations in G6PD that had not been reported previously in China. Three of these were exonic missense mutations: c.682G>A/p.D228N, c.479G>A/p.S160N, and c.404A>T/p.N135I (**Figures 1A–C**). Additionally, an intron variation not previously reported in the Chinese population, c.486-7C>G in G6PD intron 5, was identified (**Figure 1D**).

Among the mutations, 16 were male hemizygous, four were female heterozygous, and one was female homozygous; 81.25% of the G6PD mutations were located in exons 12, 6, 9, and 2, with c.1388G>A, c.487G>A, c.1376G>T, c.1024C>T, and c.95A>G accounting for 75.0% of all cases. The most frequent mutation sites were c.1388G>A (27.5%), c.487G>A (20.0%), and c.1376G>T (13.75%). **Figure 1E** displays the distribution of G6PD mutation sites among the infants.

### 3.3 Bioinformatics analysis of G6PD

Following the American Society of Medical Genetics (ACMG) guidelines, the harmfulness of three newly observed G6PD mutations in the Chinese population was predicted using PolyPhen-2, PROVEAN, and MutationTaster. A PolyPhen-2 score ≤0.452 was considered benign, 0.453–0.956 was probable, and ≥0.957 was probable. Further, a PROVEAN score ≤ -2.5 is harmful, the D of MutationTaster stands for causality, and the P stands for polymorphism; disease was likely if two of the three were predicted to be harmful. PolyPhen-2 software predicted that c.682G>A, c.479G>A, and c.404A>T had scores of 0.017, 0.997, and 0.993, respectively. PROVEAN predicted that the scores for c.682G>A, c.479G>A, and c.404A>T would be -3.257, -5.609, and -3.481, respectively. MutationTaster software predicted that the results for c.682G>A, c.479G>A, and c.404A>T were D, P, and D, respectively. Therefore, the c.682G>A, c.479G>A, and c.404A>T mutations were all predicted to be harmful.

**Figure 2:**
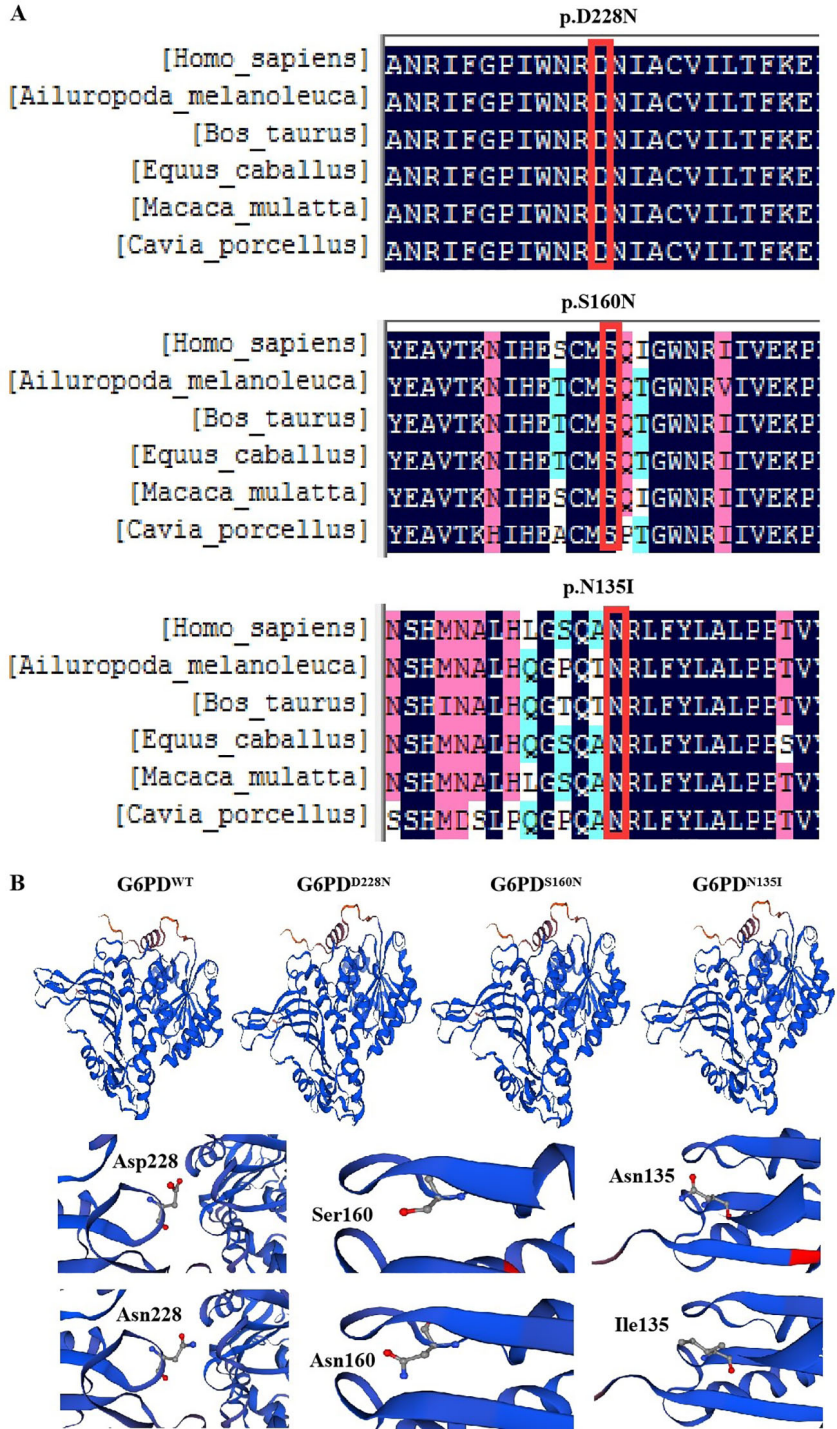
Bioinformatics analysis of G6PD. **(A)** Conservation analysis of G6PD proteins in different species. **(B)** Three-dimensional structural analysis of mutant proteins. G6PD, glucose-6-phosphate dehydrogenase.

The G6PD protein sequences from six mammals (humans, Ailuropoda melanoleuca, Bos taurus, Equus cabullus, Macaca mulata, and Cavia porcellus) were obtained from the NCBI website and compared. Conservation analysis showed that D228, S160, and N135 were located in highly conserved regions (**Figure 2A**).

We obtained the G6PD protein sequence from NCBI and constructed three-dimensional models of WT and mutant G6PD proteins using SWISS-MODEL. The comparison revealed no significant changes in the overall G6PD protein sequence. The WT protein (SMTL ID: 7sni.1.A) had 100% sequence similarity, a global model quality evaluation (GMQE) of 0.93, and a QMEAN of -0.37. The G6PD^D228N^ sequence showed 99.86% sequence similarity, with a GMQE of 0.93 and a QMEAN of -0.38. The G6PD^S160N^ sequence had 99.61% similarity, a GMQE of 0.93, and a QMEAN of -0.31. The G6PD^N135I^ sequence had 99.71% similarity, a GMQE of 0.93, and a QMEAN of -0.38. Significant changes between the mutants and WT were observed in the protein structures of G6PD^D228N^, G6PD^S160N^, and G6PD^N135I^ (**Figure 2B**).

### 3.4 Pathogenicity of the novel G6PD mutational sites c.479G>A/p.S160N and c.404A>T/p.N135I

**Figure 3:**
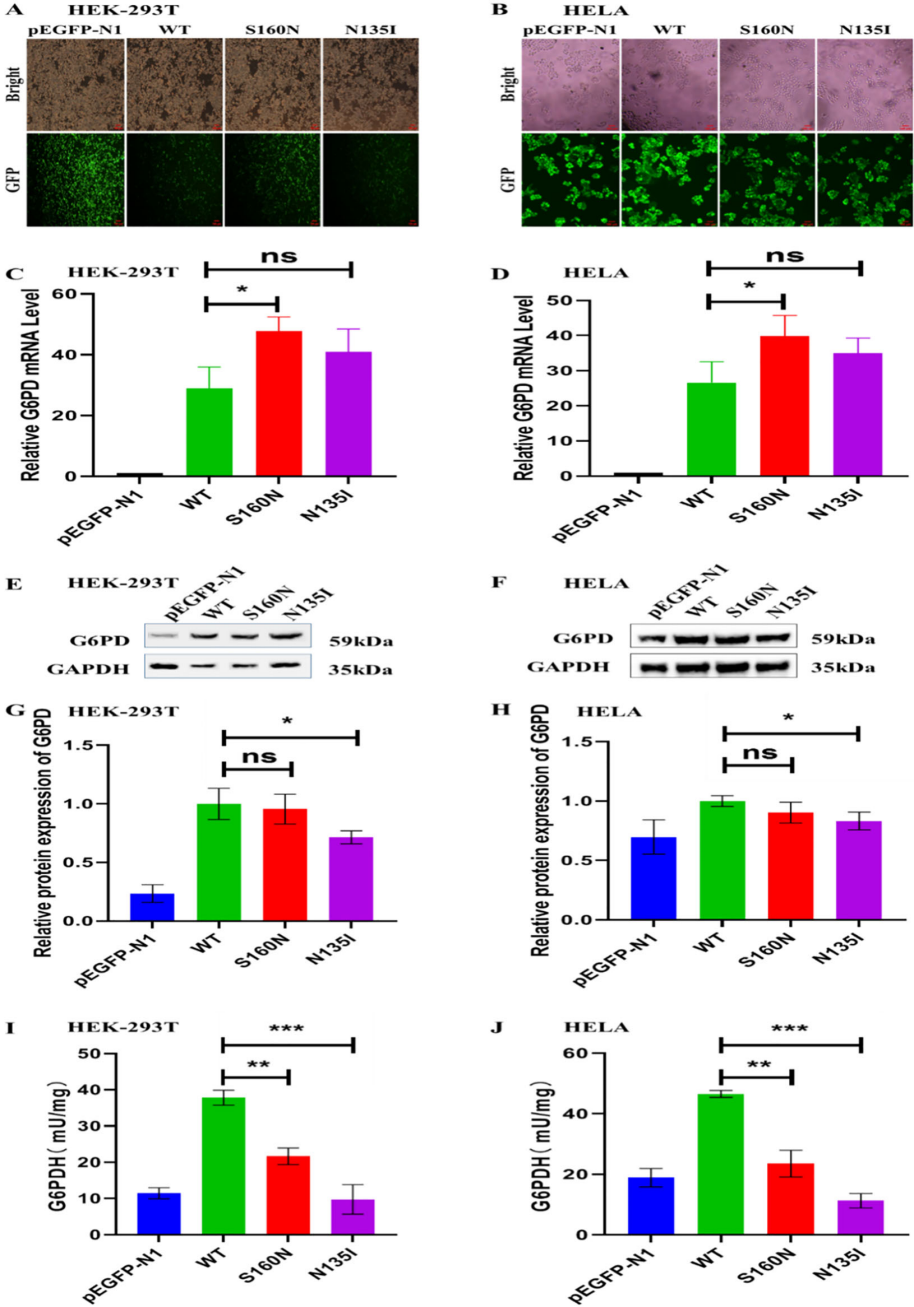
The pathogenicity of new mutation sites c.479G>A/p.S160N and c.404A>T/p.N135I in G6PD in HEK-293T and HELA cells. **(A, B)** Green fluorescent protein expression in HEK-293T and HELA cells after 24 h of plasmid transfection. **(C, D)** qPCR detection of the relative G6PD mRNA levels in different transfection groups. **(E–H)** Western blot analysis of the relative G6PD protein levels in different transfection groups. **(I, J)** Comparative analysis of the G6PD activity between the wild type and different mutation groups. *P<0.05; **P<0.01; ***P<0.001. G6PD, glucose-6-phosphate dehydrogenase; qPCR, quantitative polymerase chain reaction; ns, not significant.

After 24 h of plasmid transfection, the HEK-293T and HELA cells were observed using fluorescence microscopy. Green fluorescence was observed in cells transfected with empty, G6PD WT, and mutant plasmids, indicating successful plasmid transfection (**Figures 3A, B**). A high fluorescence intensity indicated high plasmid transfection efficiency. Following the 24-h transfection of HEK-293T and HELA cells, the levels of WT and mutant (S160N and N135I) G6PD were compared using pEGFP-N1 as a control (**Figures 3C, D**). Compared with the control group, G6PD RNA expression was noticeably higher in the WT group following transfection. Compared with the WT plasmid, the mutant plasmid S160N exhibited significantly greater mRNA expression (P<0.05), whereas the mutant N135I showed a non-significant increase.

Following 48-h transfection of HEK-293T and HELA cells, western blot analysis was performed using pEGFP-N1 transfectants as the control group (**Figures 3E–H**). In comparison to the group under control, the expression of G6PD in WT cells was increased. Compared with the WT group, the mutant plasmid S160N showed slightly less protein expression, even though the fact that there was no statistically significant difference, whereas the N135I mutation showed significantly decreased G6PD protein levels (P<0.05).

As shown in **Figures 3I–J**, G6PD activity decreased significantly in the S160N and N135I mutation groups than in the WT group. Consistent results observed in both cell lines indicate a notable effect of the mutations.

## 4 Discussion

In this study, we investigated the regional incidence and genetic mutation spectrum of G6PD deficiency in newborns in the Heze area. G6PD is a regulatory enzyme that controls oxidative stress in nucleated red blood cells and mitochondria (8). Severe G6PD deficiency can cause severe metabolic disorders and hemolytic diseases (9, 10). Molecular detection of G6PD deficiency can help to identify specific G6PD mutation types, and the commonly used detection methods include whole gene, exon, and common site detection. Although commercial kits can detect common mutation types, their comprehensiveness is limited (11–14).

Globally, the G6PD deficiency distribution is linked to historical malaria prevalence and geographic factors, with a greater incidence in tropical and subtropical regions (15–26%) and a lower incidence in North America and Europe (<3%) (15–17). Screening for genetic disorders related to metabolism in newborns in China began in the 1980s, and the frequency of G6PD deficiency in Chinese children was 4.03% (18). Consistent with the trend of “high in the south and low in the north,” the incidence of G6PD deficiency was greater in South and Southwest China, including Guangdong Province (3.1–16.1%) (19), Guangxi Province (7.40%) (20), Yunnan Province (7.39%) (21), and Guizhou Province (3.43%) (22). The incidence was lower in northern China, including in Qingdao in Shandong Province (0.02%) (23), Liaocheng in Shandong Province (0.02%) (24), Baoji in Shaanxi Province (0.02%) (25), and Shijiazhuang in Hebei Province (0.05%) (26). Our finding that the incidence of neonatal G6PD deficiency in Heze area, Shandong Province, was 0.07%, is consistent with the trend of lower incidence in North China. Notably, among areas in the Shandong Province, the G6PD deficiency incidence in Heze area is greater than that in Liaocheng and Qingdao.

The types of common G6PD mutations differ significantly across regions and nationalities (27–30). The main mutations in Chinese individuals are c.1388G>A, c.1376G>T, and c.95A>G (31), which are distributed among the various ethnic groups in China. In the present study, we detected 17 G6PD mutation types, among which 13 have been reported in the Chinese population. However, the detection rate of the c.487G>A mutation in the Heze area was greater than that of c.1376G>T, ranking second, suggesting that difference in population and economic and cultural development have altered the genetic mutation types of G6PD deficiencies in the Heze area.

Upon searching MasterMind, NCBI ClinVar, and the China National Genome Database, we identified three types of exon missense mutations that had not been reported in the Chinese population: c.682G>A (p.D228N, variant Coimbia, discovered by Chalvam et al. (32). in 2008), c.479G>A (p.S160N, not reported domestically or internationally), and c.404A>T (p.N135I, not reported domestically or internationally). We also found an intron variant not reported in the Chinese population: c.486-7C>G (this variant was the only case; a base substitution occurred in the first seven nucleotides of exon 6, possibly affecting the splicing of G6PD protein and pathogenic). These findings indicate the regional characteristics of deficiencies caused by G6PD mutations in newborns in the Heze area. Based on the ACMG guidelines, all three newly discovered missense mutations are predicted to be pathogenic. A sequence alignment revealed that the three mutated sites are in highly conserved regions, suggesting that they likely affect protein structure and function.

Most single-gene diseases are caused by mutations that alter the structure or number of encoded proteins (33). The thermal stability of an enzyme indicates its structural stability at high temperatures, and mutations in important amino acids may cause protein-folding abnormalities and affect thermal stability. A combination of decreased catalytic activity and thermodynamic stability caused the decrease in the activity of mutant G6PD enzymes (34). As the pathogenicity of the c.479G>A/p.S160N and c.404A>T/p.N135I mutations has not been reported, we used UCSF Chimera software and compared the WT and mutant amino acid structures to reveal changes in the amino acid side chains. The c.404A>T/p.N135I mutation was located in the β-fold on the G6PD protein surface, replacing asparagine with isoleucine and causing the loss of hydrogen bonds, which affected protein structural stability. In contrast, the c.479G>A/p.S160N mutation was situated at a tight bend between the α helix and β fold on the G6PD protein surface. This alteration replaced serine with asparagine, potentially creating spatial hindrance, impeding the connection between an α helix and β fold, and affecting the stability of the protein structure. In addition to structural changes, these two mutations may affect other physicochemical properties of G6PD, such as the pH stability and substrate affinity, including the ability to adapt to different pH conditions. Because mutations can modify the enzyme charge distribution and hydrogen bond networks, they may influence the enzyme ion state and binding pattern at different pH values, thereby compromising enzyme activity and stability.

Our experiments showed that the G6PD c.479G>A/p.S160N and c.404A>T/p.N135I mutations significantly reduced G6PD enzyme activity in both cell lines; however, we also found increased mRNA and decreased protein expression. This may have been caused by compensatory cellular regulation or a dominant-negative effect. Despite being translated, mutant proteins may not incorporate into the enzyme complex; however, it may interfere with the function of WT proteins by altering the protein structure (35). This study has limitations. The low prevalence of neonatal G6PD deficiency in Heze led to a small sample, potentially unrepresentative of the broader population. Most affected children receive symptomatic support and remain asymptomatic. Follow-up of 80 cases showed no symptoms, obscuring the genotype-phenotype relationship. As there is no cure, management involves avoiding certain foods (for example, broad beans), infections, primaquine, and some herbs, as well as seeking immediate prompt medical care for acute hemolysis. These factors limit our findings' generalizability, highlighting the need for more research on the genotype-phenotype link and therapeutic options.

In summary, the incidence of G6PD deficiency in newborns in the Heze area was relatively low, and the incidence and genetic mutations showed regional characteristics. This study discovered four new mutations in G6PD in the Chinese population. Based on the enzyme activity, G6PD c.479G>A/p.S160N and c.404A>T/p.N135I are potentially pathogenic and cause G6PD deficiency through different mechanisms.

## Data Availability

The datasets generated for this study can be found in the ScienceDB at https://doi.org/10.57760/sciencedb.13917. All data is publicly accessible via https://www.scidb.cn/en/s/vQB77f.
